# Comparative proteomics and gene expression analyses revealed responsive proteins and mechanisms for salt tolerance in chickpea genotypes

**DOI:** 10.1186/s12870-019-1793-z

**Published:** 2019-07-09

**Authors:** Mohammad Arefian, Saeedreza Vessal, Saeid Malekzadeh-Shafaroudi, Kadambot H. M. Siddique, Abdolreza Bagheri

**Affiliations:** 1 Plant Biotechnology and Breeding Department, College of Agriculture, Ferdowsi University of Mashhad, Mashhad, Iran; 2 Research Center for Plant Sciences, Ferdowsi University of Mashhad, Mashhad, Iran; 3 The UWA Institute of Agriculture, The University of Western Australia, Perth, WA 6001 Australia

**Keywords:** Chickpea, Differentially expressed proteins, Metabolic pathway, Salt-tolerance mechanism

## Abstract

**Background:**

Salinity is a major abiotic stress that limits the growth, productivity, and geographical distribution of plants. A comparative proteomics and gene expression analysis was performed to better understand salinity tolerance mechanisms in chickpea.

**Results:**

Ten days of NaCl treatments resulted in the differential expression of 364 reproducible spots in seedlings of two contrasting chickpea genotypes, Flip 97-43c (salt tolerant, T1) and Flip 97-196c (salt susceptible, S1). Notably, after 3 days of salinity, 80% of the identified proteins in T1 were upregulated, while only 41% in S2 had higher expression than the controls. The proteins were classified into eight functional categories, and three groups of co-expression profile. The second co-expressed group of proteins had higher and/or stable expression in T1, relative to S2, suggesting coordinated regulation and the importance of some processes involved in salinity acclimation. This group was mainly enriched in proteins associated with photosynthesis (39%; viz. chlorophyll a-b binding protein, oxygen-evolving enhancer protein, ATP synthase, RuBisCO subunits, carbonic anhydrase, and fructose-bisphosphate aldolase), stress responsiveness (21%; viz. heat shock 70 kDa protein, 20 kDa chaperonin, LEA-2 and ascorbate peroxidase), and protein synthesis and degradation (14%; viz. zinc metalloprotease FTSH 2 and elongation factor Tu). Thus, the levels and/or early and late responses in the activation of targeted proteins explained the variation in salinity tolerance between genotypes. Furthermore, T1 recorded more correlations between the targeted transcripts and their corresponding protein expression profiles than S2.

**Conclusions:**

This study provides insight into the proteomic basis of a salt-tolerance mechanism in chickpea, and offers unexpected and poorly understood molecular resources as reliable starting points for further dissection.

**Electronic supplementary material:**

The online version of this article (10.1186/s12870-019-1793-z) contains supplementary material, which is available to authorized users.

## Background

Salinity stress is one of the most significant constraints to plant growth and productivity as plants are unable to move or escape from this stress [1]. The metabolic imbalances triggered by osmotic stress, ion toxicity, and nutritional deficiency due to salinity can cause oxidative stress [2]. Plants resort to adaptive strategies to cope with salinity, including the accumulation of compatible osmolytes and proteins involved in stress tolerance. Increasing evidence has revealed that the modification of protein synthesis or degradation at both quantitative and qualitative levels is an essential metabolic process that critically influences salinity or dehydration tolerance [3, 4].

Numerous studies have offered insight into plant adaptive mechanisms for high productivity and stress tolerance. High throughput genetic screening techniques have identified significant cellular and molecular responses to various stresses as part of the complex gene network [5]. However, a clearer picture is needed of the tolerance mechanisms in plants. Recent studies have focused on high throughput proteomics analyses to look beyond transcriptomics, as various gene products are subject to post-translation modifications [6]. These studies have characterized the molecular basis for salinity tolerance at the proteome level, in crops such as rice [7], wheat [8], and *Medicago truncatula* [9]. While proteome research is quite advanced in these plants, there are no specific reports of this kind on chickpea (*Cicer arietinum* L.).

Chickpea is the third most important pulse legume and is grown for its rich protein grains. It is an excellent crop for natural resource management due to its symbiotic relationship with both nitrogen-fixing bacteria and arbuscular mycorrhizal fungi [10]. However, chickpea growth and development are limited by moderate to severe salinity stress [11].

The major mechanisms involved in plant adaptations to salinity are regulated by modifications in gene and protein expression [12]. However, proteomics analyses of chickpea responses to abiotic stress have been mainly assessed under dehydration at different subcellular levels—including nuclear [13, 14], membrane [15], and extracellular matrix (ECM) proteome [16]—in the leaves of 3-week-old seedlings. Recently, proteome changes in response to heat stress were studied in the leaves of two chickpea genotypes [17]. Based on these studies, the tolerance of chickpea to heat and dehydration stresses was attributed to altered expression of numerous functional proteins, especially those involved in cell defense and rescue, photosynthesis and energy metabolism, redox homeostasis, and signal transduction.

Despite the importance of chickpea and its salt sensitivity, there have been no large-scale proteomics studies undertaken in this area. The salinity response in plants is a complex phenomenon, where the exact structural and functional adjustments caused by this stress are poorly understood. Plants under saline stress have evolved sophisticated mechanisms, including specific ion uptake/exclusion, toxic ion compartmentalization, synthesis of appropriate products, increases in antioxidative enzymes, regulation of photosynthetic and energy metabolism, hormonal adjustments, and cell structure alterations [6]. The principal features of salt tolerance at the proteomics level in pea [18], soybean [19], and maize [20] are photosynthesis, proteolysis, protein biosynthesis, osmotic homeostasis, defiance, and stress-related proteins. The key proteins contributing to salt tolerance responses in these studies were ribulose-1, 5-bisphosphate carboxylase (Rubisco), heat shock proteins (HSPs), late embryogenesis abundant (LEA) proteins, antioxidative enzymes, glucose-6-phosphate 1-dehydrogenase, glycerate dehydrogenase, NADPH-producing dehydrogenase, glutamate synthase, and glutamine synthetase.

This study aimed to isolate novel salt-stress-responsive proteins regulated in chickpea leaves. The 2-DE along with LC-MS/MS analyses were used to characterize 65 differential expressed proteins (DEPs), which were classified based on their putative functions. We provide evidence for the expression of novel proteins that have never been associated with chickpea under abiotic stress or plant response to salinity. Supplementary information from the gene expression analysis was derived to better understand the molecular mechanisms of the salinity response. Overall, this study offers new insights into the molecular mechanisms of salt tolerance underlying responsive proteins and genes in chickpea genotypes.

## Results

### 2-DE maps and identification of DEPs

**Fig. 1:**
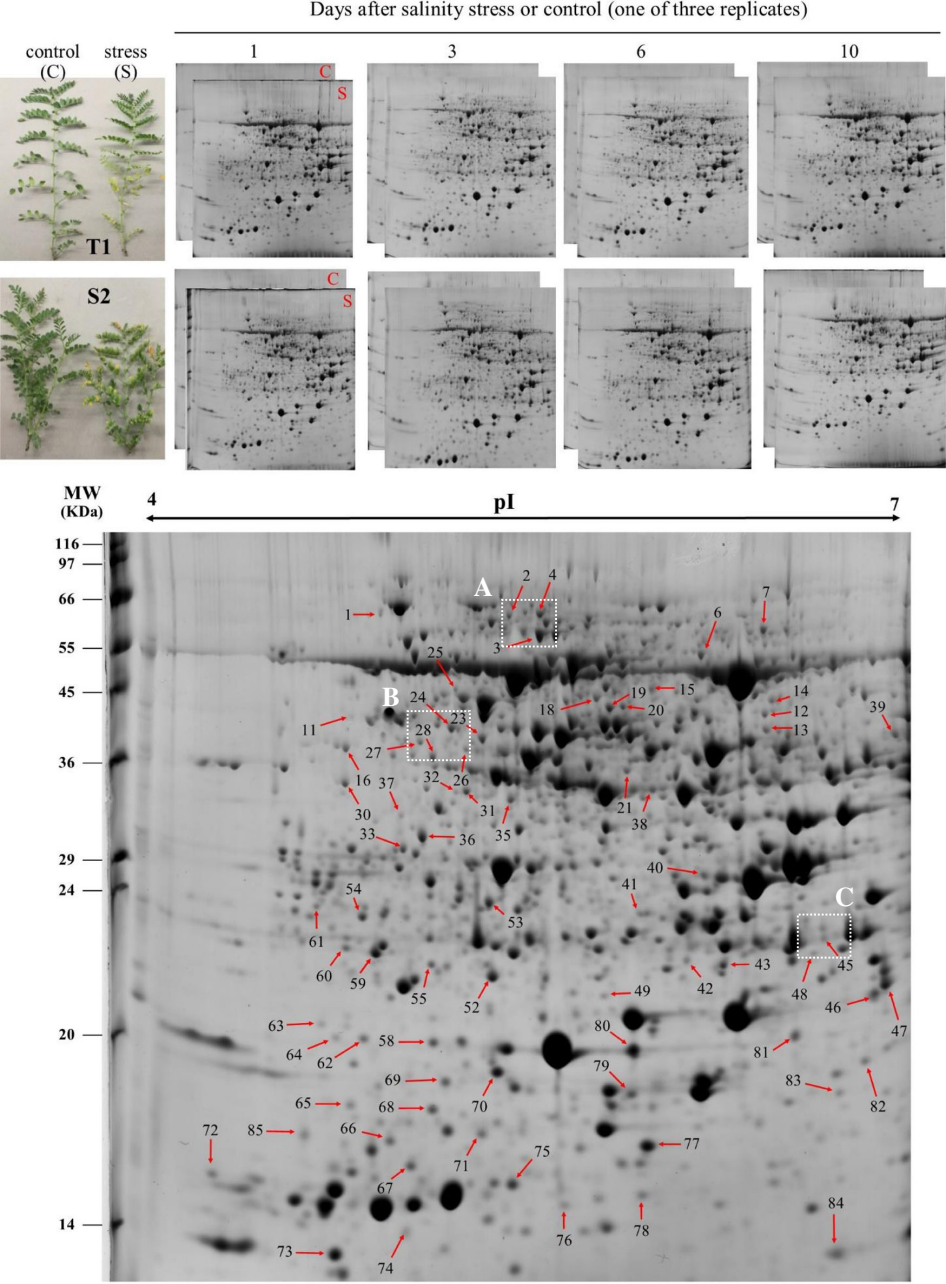
Representative 2-DE gel electrophoresis maps of leaf proteins for two chickpea genotypes after different days of salinity stress and the control. Three boxed areas (A–C) marked with dotted lines represent the zoomed in gel sections in Fig. 2. The numbers correspond with the spot ID, mentioned in Table 3

**Fig. 2:**
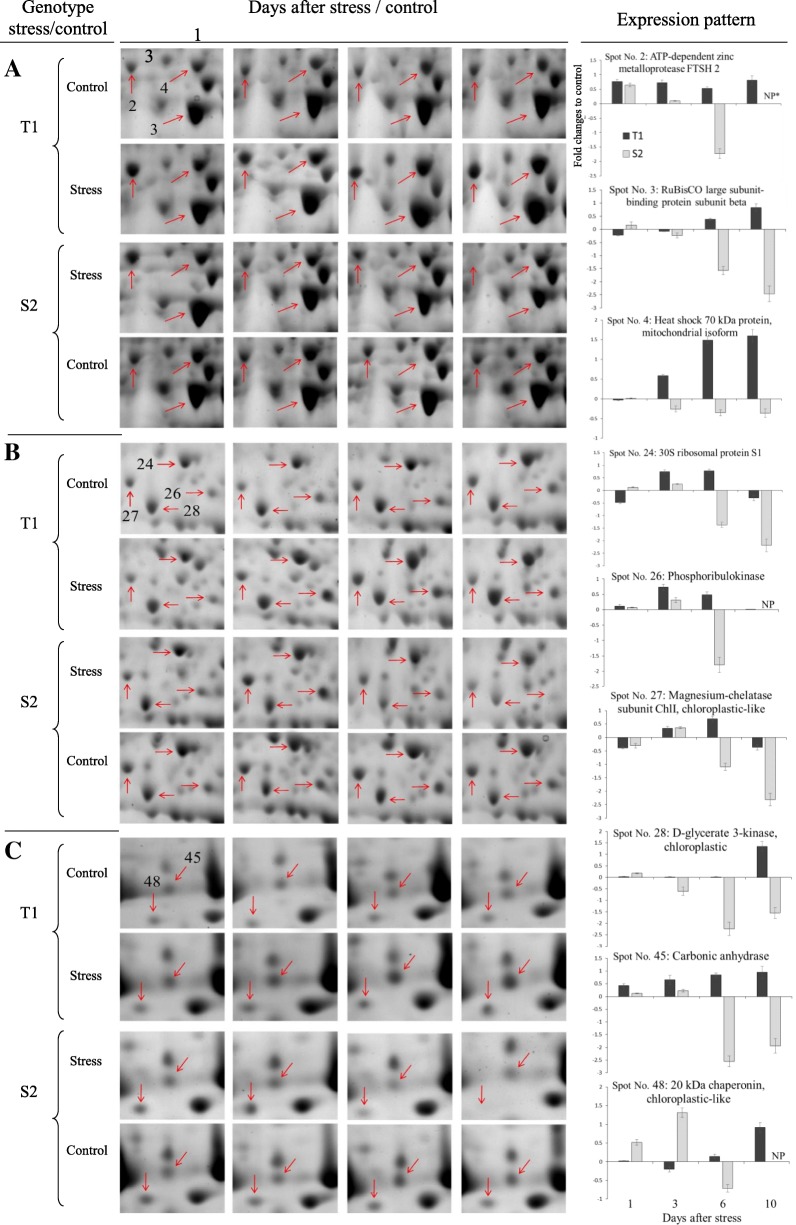
The relative expression patterns of nine representative protein spots in two chickpea genotypes under 0 and 100 mM NaCl. Boxed areas (**a**–**c**) are zoomed in gel sections, corresponding to the dotted line boxes in Fig. 1. The expression patterns in terms of fold-changes to control are presents on the right. NP* represents not present or absent spot

The 2-DE map analyses of the complete leaf of T1 and S2 seedlings exposed to 0 and 100 mM NaCl for 10 days revealed at least 1400 reproducible protein spots that were present in both genotypes and all 2-DE gels (Additional file 1: Figure S1 and Additional file 2: Figure S2). Representative 2-DE gels of the proteome of two chickpea genotypes under saline and control conditions for 10 days is presented in Fig. 1; high-resolution protein spots were obtained in the 4–7 (linear) p*I* and 6.5–200 kDa *M*_r_ range. Of these, 364 spots were classified as DEPs in at least one combination of genotype (T1 and S2) × time (1, 3, 6 and 10 DAT) in the salinity treatment (100 mM), relative to the respective control (0 mM). As a result, 65 DEPs were selected based on quantitative data (more than two fold-changes) from the Image Master analysis and visual inspection of 2-DE gels (classified as ‘high-quality’ spots with differential expression patterns during 10 days of stress). Nine spots in three typical gel regions are represented in zoom images in Fig. 2, displaying differential expression of some proteins. The LC-MS/MS analysis positively identified just over 98% of selected proteins (64 spots) with significant (*p* ≤ 0.05) hits, with an average MASCOT score of ~ 500, representing a very high rate of success identification. More than 80% of identified proteins were hit to legume species, 47 of which were matched to protein isoforms of *C. arietinum* species (Table 3). The MASCOT score, coverage percent, theoretical/experimental p*I*, and *M*_r_ are also summarized in Table 3.

In several cases, some redundancy was observed, where the same protein resolved into multiple spots in the same gel, e.g., RuBisCO, ATP synthase, heat shock proteins (HSPs), L-ascorbate peroxidase, and elongation factor Tu. These relative DEPs recorded some differences in p*I*, *M*r, or both. This could be due to their probable post-translational modification(s) (including phosphorylation, de-amidation, acetylation, glycation, and glutathionylation), multiple isoforms, or in some cases these proteins could be degraded products, possibly from extraction, as evident in the gel-based size [3].

Generally, downregulation was the dominant trend for protein expression in chickpea in response to salinity (Table 1). Almost half of the 512 spots (two genotypes × four sampling times × 64 DEPs) were downregulated, especially S2 at 6 and 10 DAT. However, T1 at 3 and 6 DAT recorded the most spots with upregulation. Sixty-four DEPs, identified with a significant match, were considered for further functional clustering and expression profile across genotype and time of salinity.

**Table 1:** Comparison of salt-responsive DEPs in the leaves of two contrasting chickpea genotypes (T1 and S2) during 1, 3, 5, and 10 days of 100 mM NaCl stress

Ex. pattern	Genotype	Days after salinity treatment				Total	
		1	3	6	10		
* Up	T1	24	51	35	21	131	189
	S2	24	26	2	6	58	
** Un	T1	12	6	17	2	37	76
	S2	18	16	2	3	39	
*** Down	T1	28	7	12	41	88	247
	S2	22	22	60	55	159	

*Up, up-regulated DEPs under salinity stress; **Un, DEPs showing no significant (*p* ≤ 0.05) response to salinity in at least one time point; *** Down, down-regulated DEPs under salinity

### Functional classification of DEPs

**Fig. 3:**
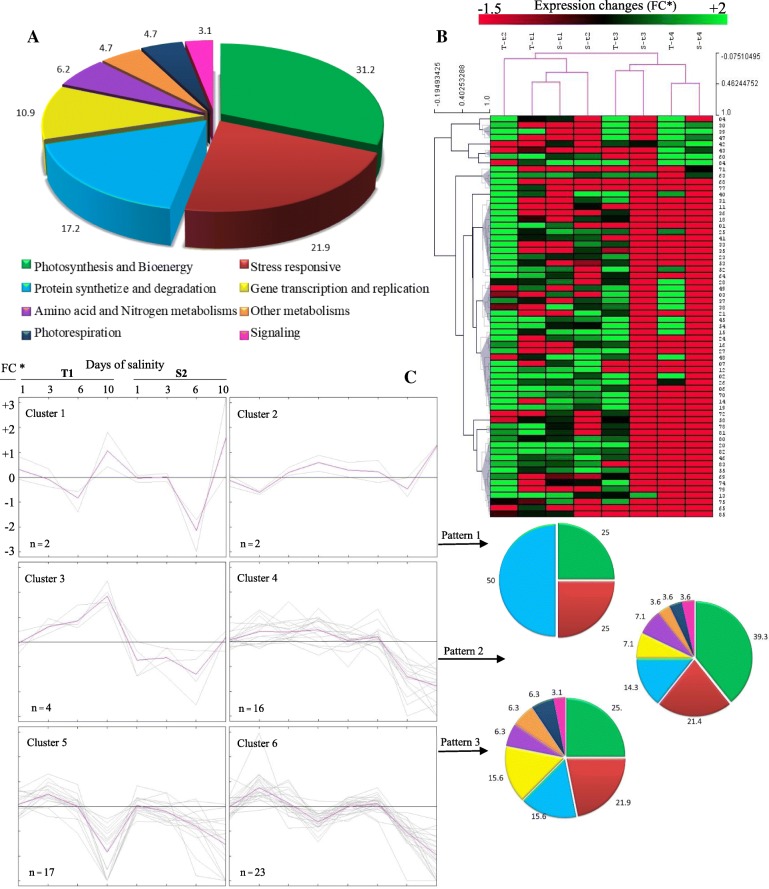
**a** An outline of functional classification for 64 differentially expressed proteins identified in the seedling leaves of two chickpea genotypes under salinity. This classification is according to the assignment of protein putative function(s) by searching in UniProt, NCBI, and associated literature; **b** Self-organizing tree algorithm (SOTA) clustering analysis of expression profiles for identified proteins under salinity for 1, 3, 5 and 10 days (t1, t2, t3, and t4, respectively); **c** Proteins were classified into six clusters and three patterns, based on their expression profiles, listed in Additional file 3: Table S1. The gray lines represent the expression profile of each protein, while the mean expression profile is marked in pink for each cluster. The expression change is based on fold-changes over respective controls (FC *)

The 64 identified protein spots were grouped into eight functional categories (Fig. 3a and Table 3) according to their assignment of putative biological function(s) by searching InterPro or associated literature, with an emphasis on the features related to salinity or dehydration responses in seedlings of chickpea, pulses, or other model plants such as Arabidopsis.

The functional analysis (Fig. 3a) indicated that ‘photosynthesis and bioenergy’ represented some 31% of the total protein spots, with ~ 22% related to ‘stress responsiveness’, which reflects the role of energy production and defense strategies within the two genotypes. Other categories included ‘protein synthesis and degradation’ (17.2%), followed by ‘gene transcription and replication’ (10.9%), ‘amino acid and nitrogen metabolism’ (6.2%), ‘photorespiration’ (4.7%), ‘signaling’ (3.1%), and ‘other metabolisms’ (4.7%). Furthermore, DEPs were classified based on their expression patterns (Fig. 3c and Additional file 3: Table S1).

### Dynamics of DEP networks

To summarize the comprehensive overview of dynamic profiles and coordinated regulation of DEPs, SOTA analysis was undertaken using fold-expression-changes data relative to the control. The analysis yielded six distinct expression clusters based on similar expression profiles for both genotypes during 10 days of salinity (Fig. 3c). The most abundant group appeared in Clusters 4, 5 and 6 only, containing proteins involved in photosynthesis and bioenergy (18 spots), stress responsiveness (11 proteins), protein synthesis and degradation (9 proteins), and gene transcription and replication (7 proteins) (Additional file 3: Table S1). Three major patterns of expression were detected (Fig. 3c and Additional file 3: Table S1):

1. Clusters 1 and 2 had an initial downregulation of DEPs, which were upregulated during later stages, especially at 10 DAT. These proteins were mainly involved in protein synthesis and degradation (50%).
2. Clusters 3 and 4 had higher expression in T1, compared with little or no change in S2, relative to their respective controls. These groups were enriched in proteins involved in photosynthesis and bioenergy (39%), stress responsiveness (21%), and protein synthesis and degradation (14%). The upregulation and/or stable expression of these DEPs may be responsible for the higher salinity tolerance in T1 than S2.
3. Clusters 5 and 6 had upregulation of DEPs in the early days of stress (3 DAT), especially in T1, which were downregulated or not expressed at later stages (10 DAT), particularly in S2. This cluster is enriched in proteins associated with photosynthesis and bioenergy (25%), stress responsiveness (22%), and protein synthesis and degradation (16%).

Figure 3b shows the results of the further analysis of SOTA to cluster the overall response of both genotypes (T1 and S2) based on their expression profile for all 64 DEPs over four time-points (t1 to t4). Both genotypes behaved similarly and clustered together at all time-points, except for 3 DAT (t2) where large differences in protein expression level were observed between the genotypes, with T1 upregulated in 51 protein spots compared with 22 in S2. In terms of the overall expression pattern of proteins across all time-points, two main clusters were formed (Fig. 3b):

1. Cluster 1 had four members with upregulation at early stages of salinity (t1 and t2) in both genotypes, with the highest expression being 80% DEPs in T-t2 (T1 genotype at 3 DAT as third time-point of stress).
2. Cluster 2 showed downregulation of DEPs as salinity progressed, with more than 95% DEPs downregulated in S-t3, followed by 86% in S-t4 (both sensitive genotype); less downregulation occurred at the corresponding time-points in the tolerant genotype.

The critical difference between the two genotypes could be related to the 14 proteins that were upregulated in T1 at 10 DAT (T-t4). These DEPs could be responsible for the higher salinity tolerance in T1 over S2, specifically at later stages in the salinity treatment.

Generally, the cellular defense mechanism seems to depend on the progress of salinity. Therefore, both early and late responses could be seen. The fact that the studied critical proteins were identified as salinity-responsive cases gives the first functional annotation to their genes and possible role in salinity response, so further investigation was performed as a complementary and conforming experiment at the transcript level.

### Comparison between proteins and transcripts

**Fig. 4:**
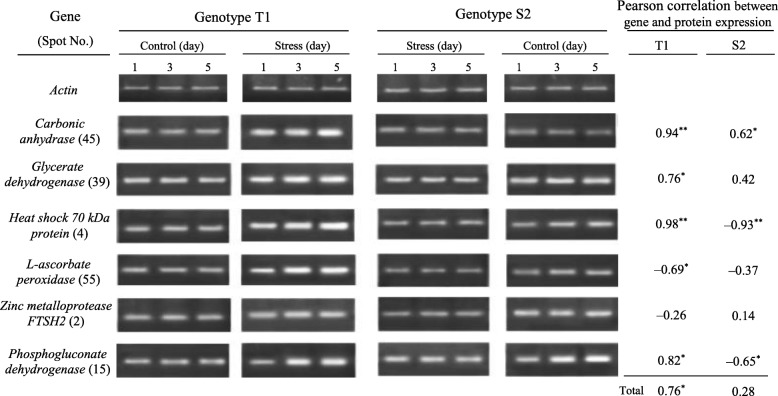
Expression profile of genes encoding six selected DEPs in salt-stressed chickpea genotypes. Transcript level was determined by RT-PCR after normalization to the actin gene at three time-points: 1, 3, and 5 days of salt stress relative to the control using three individual replicates. Correlation of gene expression with corresponding protein abundance using Pearson’s correlation analyses was calculated (* *p* ≤ 0.05, ** *p* ≤ 0.01)

Six representative leaf DEPs were used to further characterize patterns of gene expression by RT-PCR (Table 2). The choice of these genes was based on their possible biological functions during salinity stress, and their differential proteomics-derived patterns between two genotypes with emphasis on upregulation in T1 and downregulation in S2 (Clusters 3 and 4). The expression of transcripts was calculated as fold-changes relative to their respective controls (Fig. 4 and Additional file 5: Figure S4). Trends were compared to respective protein profiles using Pearson correlation analysis (Fig. 4).

**Table 2:** Selected genes for transcript profiling based on biological function and expression profiles of corresponding proteins

Gene	Biological function	Spot no. /cluster	Primer sequence (5′ – 3′)	PCR product size (bp)	GeneBank accession no.
*Actin*	Reference gene	–	F:TGTTCCCCGGAATTGCTGATAGAATGAGC R:TTGGAAAGTGCTGAGAGATGCCAAAATGGAG	146	AJ012685
*Carbonic anhydrase*	Photosynthesis	45 / 4	F: TTGAAGTGAAGGAAACC R: AGAAGAAATGGGAAAGG	195	LOC101498889
*Glycerate dehydrogenase*	Amino acid & nitrogen metabolism	39 / 3	F: GAAAGACTCGCCAAGA R: GGGCTCATCCTCAAAC	141	LOC101496862
*Heat shock 70 kDa protein*	Stress responsive	4 / 3	F: CTTGATGTAACGCCACTT R: GTCCACCAGATGACCTAATA	346	LOC101502051
*L-ascorbate peroxidase*	Stress responsive	55 / 5	F: AAATCTTACCCAACCGTCA R: CAACAACACCACCCAACT	304	LOC101497640
*ATP-dependent zinc metalloprotease FTSH2*	Protein synthesis and degradation	2 / 4	F: AAAGAACAACCGTGAAGCAA R: CGGCGACTGGTAATGGA	152	LOC101512859
*6-phosphogluconate dehydrogenase*	Other metabolisms: PP	15 / 4	F: TGACAGCAAGGCAAACAACTC R: CAGGCATACGACAGAAACCC	112	LOC101509898

There was a significant (*p* ≤ 0.05) correlation between protein abundance and RT-PCR signal for five genes (45, 39, 4, 55, and 15) in salt-stressed T1 and three genes (4, 15, and 45) in salt-stressed S2. Two proteins in T1 (55 and 2) and three proteins in S2 (4, 55, and 15) behaved against their mRNA changes during salinity stress. The calculated Pearson’s correlation was 0.76 and 0.28 in T1 and S2, respectively, which implies that the correlation between transcripts and proteins was significantly (*p* ≤ 0.05) higher in T1 than S2.

## Discussion

Plant response to salinity is a complex process that requires changes in gene and protein expression profiles. This study aimed to better understand salinity tolerance mechanisms in chickpea. The data revealed significant differences in protein and gene expression patterns between two contrasting chickpea genotypes (T1 and S2) under salinity stress (100 mM NaCl) for 10 days. This is the first comprehensive proteome analysis of chickpea under saline conditions.

### Role of photosynthesis and bioenergy in salt tolerance

**Fig. 5:**
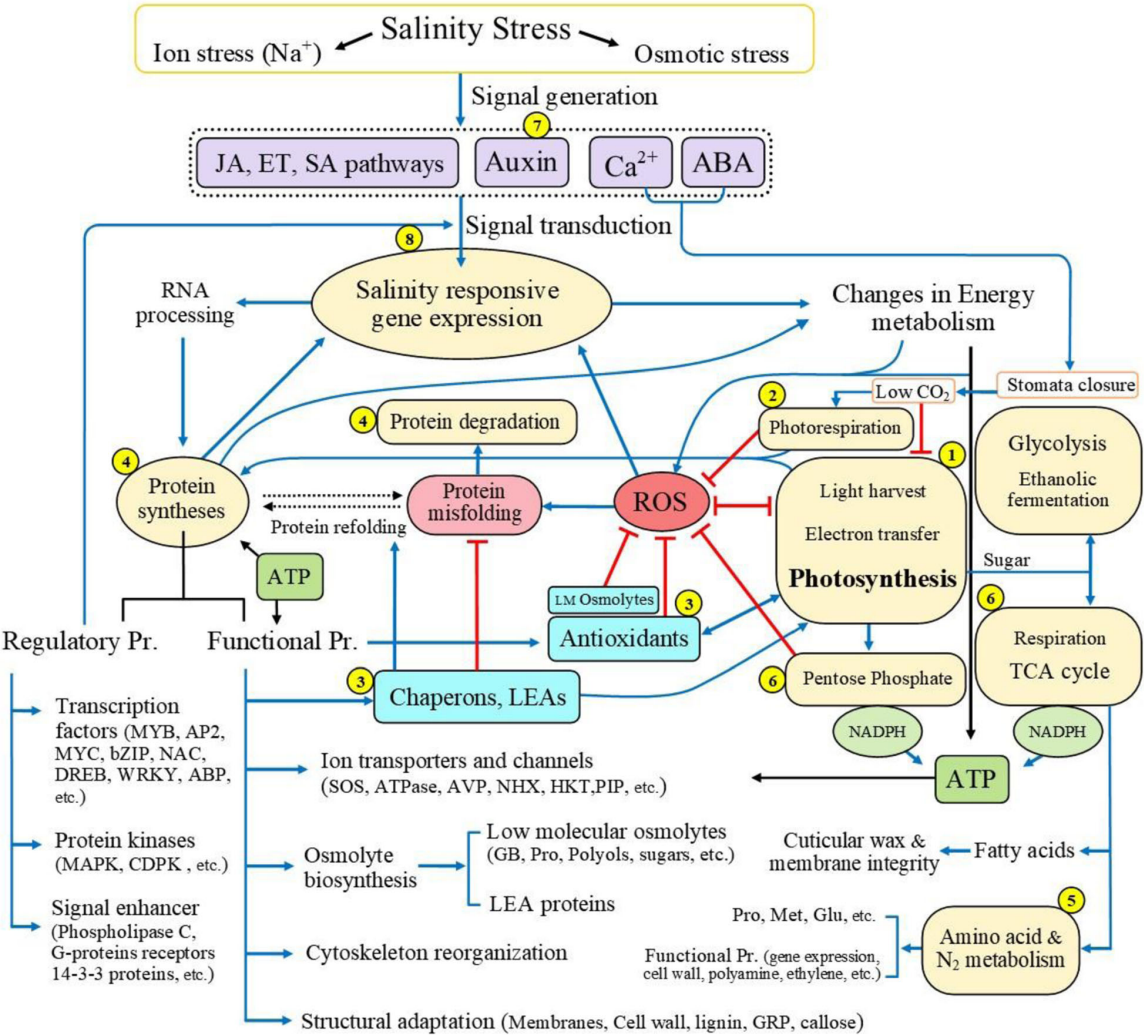
Schematic outline of the salinity-induced response pathway based on proteome, gene expression, and physiological changes in chickpea leaves. Numbers in yellow circles are corresponding to the protein(s) mentioned in Table 2. Blue arrows indicate induction or increase; red arrows indicate repression or decrease, and dotted arrows indicate possible or reversible alterations. Salinity activates several signaling cascades, regulates gene expression, and promotes regulatory and functional protein biosynthesis, in which the main role of ROS, antioxidants, and chaperones under saline condition is presented. Tolerant seedlings inhibited or decreased major metabolic pathways (i.e., photosynthesis, photorespiration, TCA cycle, and amino acid metabolism) less than susceptible seedlings to provide more energy and other compatible metabolites. Importantly, salt tolerance relies on higher transporter activities to remove H_2_O_2_, Na^+^, etc., and enhance osmotic regulation, and membrane and cell wall re-modulation to cope with the stress

Salinity changes the integrity and functionality of chloroplasts, which in turn impacts cell function as a whole [21]. In the present study, salinity had the most significant effect on the abundance of the functional group of proteins related to light-harvesting complexes, electron transfer pathway, and photosynthetic Calvin-Benson Cycle. The enzymes in this group provide substrates for the synthesis of ATP, NADPH, sucrose, starch, and proteins (Fig. 5), which improve plant biomass, yield, and resistance to stresses [22].

Five identified DEPs (49, 52, 74, 68, and 69) participated in the light-harvesting reaction and photosystems. Spots 49 and 52 were identified as the psbP domain-containing protein 1 (PPD1) and chlorophyll a-b binding protein 3 (CAB) were upregulated in T1, more so at 10 and 3 DAT, respectively, but downregulated in S2 at the corresponding time-points (Table 3). The increased CAB and PPD1 in T1, which clustered together (Fig. 2c and Additional file 3: Table S1, Cluster 4), transferred more excitation energy to the reaction center, where the accumulated plastocyanin can donate more electrons to photosystem I (PSI) to reduce NADP^+^ to NADPH [23]. Moreover, PPD1, a nuclear-encoded and thylakoid luminal protein, is an essential factor for PSI [24] and PSII [25] assembly and activity. The initial upregulation (0–12 h) and subsequent downregulation (144 h) of CAB and PPD1 in response to salinity have also been recorded in soybean genotypes [23].

**Table 3:** Details of the identified 2-DE-resolved exclusive DEPs by LC MS/MS in the leaves of tolerant (Flip 97-43c; black columns) versus susceptible (Flip 97-196c; gray columns) chickpea seedlings treated with 100 mM NaCl stress for 1, 3, 6, and 10 days. The key potential proteins contributing to the differential response of the two genotypes to salinity are indicated by * next to their spot numbers

Spot no.	Identified protein	Ac. No. ^a^	Species	MASCOT score	% Coverage (matches) ^b^	TMr/ EMr ^c^	TpI/ EpI ^d^	Time kinetics (DAT)^e^			
								1	3	6	10
	1. Photosynthesis and bioenergy										
3*	RuBisCO large subunit-binding protein subunit beta	XP_012567814.1	*Cicer arietinum*	1137	44 (93)	62.9/59.2	5.85/5.53				
70	RuBisCO large subunit, partial (chloroplast) 2	ALB07273.1	*Acanthus ebracteatus*	71	32 (15)	14.4/17.5	9.39/5.37				
84	Ribulose bisphosphate carboxylase small chain	Q9ZP07	*Cicer arietinum*	270	29 (64)	20.4/9.25	9.03/6.72				
18	ATP synthase beta subunit, partial (chloroplast)	AAK72753.1	*Cucurbita pepo*	925	38 (128)	51.9/45.2	5.04/5.76				
33	ATP synthase subunit beta, chloroplastic	B5LMK9	*Cicer arietinum*	585	39 (58)	52.9/28.7	5.16/4.99				
53*	ATP synthase subunit alpha	A0A023HPK1	*Trifolium aureum*	3840	27 (149)	55.8/24.1	5.16/5.33				
49*	psbP domain-containing protein 1, chloroplastic	XP_004494530.1	*Cicer arietinum*	643	65 (31)	28.6/20.5	8.89/5.79				
52*	Chlorophyll a-b binding protein 3, chloroplastic	XP_004491629.1	*Cicer arietinum*	244	25 (55)	29.4/21.3	3.32/5.34				
64*	Oxygen-evolving enhancer protein 1, chloroplastic	XP_004509219.1	*Cicer arietinum*	214	35 (37)	34.9/19.0	6.24/4.72				
79	Oxygen-evolving enhancer protein 2, chloroplastic	XP_004499534.1	*Cicer arietinum*	537	32 (60)	28.7/16.5	6.90/5.90				
74	Photosystem I reaction center subunit II, chloroplastic-like	XP_004511355.1	*Cicer arietinum*	625	34 (37)	23.7/12.5	9.66/5.00				
68	2Fe-2S iron-sulfur cluster-binding domain protein	G7IUN0	*Medicago truncatula*	70	8 (1)	21.5/15.7	6.82/5.11				
69	Cytochrome b6-f complex iron-sulfur subunit	A0A067FP73	*Gossypium raimondii*	61	13 (4)	32.6/16.7	8.48/5.16				
27	Magnesium-chelatase subunit ChlI, chloroplastic-like	XP_004485870.1	*Cicer arietinum*	244	34 (32)	45.4/39.5	5.32/5.06				
26	Phosphoribulokinase, chloroplastic	XP_004512451.1	*Cicer arietinum*	369	30 (28)	45.5/38.8	6.41/5.24				
45*	Carbonic anhydrase, chloroplastic isoform X2	XP_004489275.1	*Cicer arietinum*	409	19 (36)	35.8/22.3	6.61/6.66				
38*	Fructose-bisphosphate aldolase 1, chloroplastic	XP_004507507.1	*Cicer arietinum*	1376	21 (68)	43.2/33.5	6.28/5.98				
63	Phosphoglycerate kinase, cytosolic-like	XP_004488763.1	*Trifolium aureum*	1452	35 (121)	49.8/19.2	7.79/4.66				
78	Transketolase, chloroplastic	XP_012570278.1	*Cicer arietinum*	605	18 (25)	79.9/13.5	6.00/5.95				
	2. Photorespiration										
7	Glycine dehydrogenase/decarboxylating, mitochondrial	XP_004498896.2	*Cicer arietinum*	653	26 (42)	115.3/60.2	7.99/6.43				
21	Glutamate--glyoxylate aminotransferase	XP_004489787.1	*Cicer arietinum*	359	33 (28)	53.3/35.7	5.83/5.88				
28*	D-glycerate 3-kinase, chloroplastic	XP_004486714.1	*Cicer arietinum*	116	19 (12)	46.4/37.2	5.39/5.11				
	3. Stress responsive										
1	70 kDa heat shock protein	Q1SKX2	*Medicago truncatula*	153	11 (12)	75.7/64.7	5.19/4.96				
4*	Heat shock 70 kDa protein, mitochondrial isoform	XP_012572445.1	*Cicer arietinum*	833	40 (67)	72.1/64.7	5.70/5.54				
11	Heat shock protein 70 kDa	A0A0A1HAD2	*Chrysanthemum morifolium*	265	13 (55)	70.8/42.2	5.12/4.86				
58	Heat shock 22 kDa protein, mitochondrial isoform X1	XP_004506342.1	*Cicer arietinum*	249	25 (22)	25.1/18.75	6.78/5.11				
81	Cold shock protein	A0A088FZS5	*Cicer arietinum*	1497	31 (51)	19.2/18.5	6.29/6.55				
48*	20 kDa chaperonin, chloroplastic-like	XP_004508023.1	*Cicer arietinum*	413	50 (17)	26.7/21.5	9.05/6.63				
30*	uncharacterized protein, Homologues with LEA-2	XP_004513216.1	*Cicer arietinum*	400	59 (49)	34.3/35.0	4.63/ 4.76				
31	Thiamine thiazole synthase, chloroplastic	I3TAU0	*Lotus japonicus*	133	17 (12)	37.7/33.2	5.02/5.24				
40	Xanthoxin dehydrogenase	XP_004499827.1	*Cicer arietinum*	573	41 (49)	29.6/28.3	5.89/ 6.19				
42*	Glutathione s-transferase	A0A0X9LEN0	*Cicer arietinum*	270	34 (38)	25.6/21.8	6.04/6.12				
41*	L-ascorbate peroxidase, cytosolic	XP_004505943.1	*Cicer arietinum*	243	37 (49)	27.1/23.2	5.65/ 5.94				
55	L-ascorbate peroxidase, cytosolic	XP_004505943.1	*Cicer arietinum*	501	81 (67)	27.1/21.5	5.65/5.10				
77	Superoxide dismutase [Cu-Zn]	A0A0V0HK97	*Solanum chacoense*	1057	15 (72)	22.4/14.5	6.08/5.96				
83	Apolipoprotein D-like	XP_004507714.1	*Cicer arietinum*	128	35 (29)	21.3/16.5	6.84/6.73				
	4. Protein synthesis and degradation										
2*	ATP-dependent zinc metalloprotease FTSH 2	XP_004504668.1	*Cicer arietinum*	945	36 (74)	74.6/62.7	5.60/5.34				
6	Probable mitochondrial-processing peptidase subunit beta	XP_004492264.1	*Cicer arietinum*	520	41 (56)	59.4/54.5	6.22/6.18				
60	Metacaspase-4-like	XP_004510001.1	*Cicer arietinum*	158	10 (14)	45.2/22.0	5.08/4.76				
25	26S protease regulatory subunit 6A homolog	XP_004495708.1	*Cicer arietinum*	382	50 (29)	47.4/47.0	4.98/5.20				
43	Proteasome subunit beta type	A0A151TBQ9	*Cajanus cajan*	68	21 (10)	24.6/21.5	6.89/6.27				
13	Elongation factor Tu, mitochondrial	XP_004493639.2	*Cicer arietinum*	272	30 (54)	49.1/40.2	6.58/6.44				
20	Elongation factor Tu, chloroplastic	XP_004501869.1	*Cicer arietinum*	388	30 (33)	53.5/44.0	6.25/5.87				
23*	Elongation factor Tu, chloroplastic	XP_004501869.1	*Cicer arietinum*	562	29 (52)	53.5/39.2	6.25/5.31				
24	30S ribosomal protein S1, chloroplastic	XP_004486520.1	*Cicer arietinum*	721	64 (48)	44.5/40.7	5.33/5.18				
72	60S acidic ribosomal protein P3-like	XP_004504130.1	*Cicer arietinum*	420	38 (30)	12.1/14.2	4.20/4.23				
85	50S ribosomal protein L9, chloroplastic	XP_004506080.1	*Cicer arietinum*	27	9 (3)	22.4/15.7	9.78/4.6				
16*	peptidyl-prolyl cis-trans isomerase CYP38, chloroplastic	XP_004489294.1	*Cicer arietinum*	628	43 (68)	50.1/38.5	5.12/4.76				
	5. Amino acid and nitrogen metabolism										
19	S-adenosylmethionine synthase	C3TS15	*Cicer arietinum*	462	50 (61)	43.3/43.7	5.50/5.81				
35	Glutamine synthetase leaf isozyme, chloroplastic	Q9XQ94	*Medicago sativa*	65	14 (30)	47.1/33.0	6.29/5.42				
39*	Glycerate dehydrogenase	XP_004497175.1	*Cicer arietinum*	963	36 (80)	42.2/39.2	6.62/6.95				
46	Hydroxyacylglutathione hydrolase 2, mitochondrial-like isoform X	XP_004486896.1	*Cicer arietinum*	20	5 (2)	36.7/21.3	9.03/6.87				
	6. Other metabolisms (TCA, PP, and purine)										
12	Isocitrate dehydrogenase [NADP]	G7KFV7	*Medicago truncatula*	137	14 (39)	45.9/42.7	5.99/6.43				
15	6-phosphogluconate dehydrogenase, decarboxylating 3	XP_004491970.2	*Cicer arietinum*	524	43 (77)	53.6/47.2	5.88/5.99				
36	Phosphoribosylformylglycinamidine cyclo-ligase, chloroplastic/mitochondrial-like	XP_004500242.1	*Cicer arietinum*	131	18 (14)	41.1/29.7	5.65/5.06				
	7. Signaling										
47*	Auxin-binding protein ABP19a-like	XP_004513480.1	*Cicer arietinum*	569	28 (46)	21.9/20.7	6.95/6.91				
80*	Low molecular weight phosphotyrosine protein phosphatase	XP_004506147.1	*Cicer arietinum*	287	12 (12)	27.1/17.7	7.62/5.9				
	8. Gene transcription and replication										
82	Nascent polypeptide-associated complex subunit beta	B7FMW7	*Medicago truncatula*	327	42 (24)	17.5/17.5	6.75/6.83				
14	Polyadenylate-binding protein RBP45B isoform X2	XP_012573405.1	*Cicer arietinum*	277	16 (14)	34.8/44.7	6.31/6.46				
37*	33 kDa ribonucleoprotein, chloroplastic	A0A0B2SU82	*Glycine soja*	66	3 (2)	30.4/31.5	8.67/4.98				
54*	29 kDa ribonucleoprotein A, chloroplastic	XP_004497514.1	*Cicer arietinum*	110	16 (5)	30.7/23.2	5.36/4.83				
65	LOC101493535 isoform X1, homologous with smad/FHA domain protein	XP_004502003.1	*Cicer arietinum*	1469	35 (93)	24.3/16.5	5.69/4.78				
71	Histone H2B	A0A0K9PL95	*Zostera marina*	109	20 (18)	15.5/15.3	10.05/5.3				
75	Glycine-rich RNA-binding protein-like	XP_004507449.1	*Cicer arietinum*	252	22 (27)	16.1/13.5	6.32/5.43				

^a^Accession numbers correspond to NCBI or Uniprot entries

^b^Percentage of the protein sequence covered by matching peptides and number of matched peptides in the database

^c^Theoretical molecular weight (*M*_r_) and p*I*, predicted from MS/MS analysis

^d^Experimental molecular weight (*M*_r_) and p*I* were estimated using standard protein markers and automatic assignment by image analyzer (Image Master) software

^e^Protein expression profile of T1 (dark columns) and S2 (light columns) genotype relative to control, shown as horizontal axis

Two protein spots were identified as chloroplastic oxygen-evolving enhancer protein (OEE) 1 (spot 64) and 2 (spot 79). The upregulation of both OEEs in T1, relative to S2, mirror greater efficiency in water-splitting and PSII core assembly/stability [25] in the first 6 days of stress (Table 3). Salt-stress treatments have paradoxical impacts on OEE; for instance, salinity causes its upregulation in *Triticum aestivum* [26] and halophyte *Halogeton glomeratus* [27], but downregulation in *Spinacia oleracea* [25].

Three DEPs were identified as subunits of ATP synthase—alpha (spot 53) and beta (spots 18 and 33)—which participate in photosynthesis. These proteins (Fig. 3c, Cluster 4) were markedly (*p* ≤ 0.05) upregulated at 3 DAT in T1, but significantly (*p* ≤ 0.05) downregulated at later stages of salt stress (6 and 10 DAT) in the seedling leaves of S2. These results suggest that energy synthesis is inhibited in salt-stressed S2 seedlings at late-stress stages, and the synthesis of ATP correlates well with light-harvesting and electron transfer proteins. ATP-dependent synthase/protease could have chaperone-like activity [28], which is a crucial coping strategy for plants under salt stress [29]. In addition, the increased abundance of this complex in T1 under salt stress has an indirect role in the translocation of excess Na^+^ and Cl^−^ ions into the vacuole [30]. The increased ATP supply in T1 seems to meet the increased stress-related energy demand to increase Calvin cycle efficiency. Consistent with our results, the higher expression of this enzyme in tolerant than susceptible genotypes under salinity or dehydration stress has been reported in barley [27] and common bean [31]. In contrast, ATP synthase expression was significantly downregulated in a tolerant cowpea cultivar, but upregulated in a sensitive one, after salinity stress [32].

Eight proteins were engaged in carbon assimilation: carbonic anhydrase (CA, spot 45), phosphoribulokinase (PRK, spot 26), ribulose 1,5-bisphosphate carboxylase/oxygenase (RuBisCO) large (spots 3 and 70) and small (spot 84) subunits, phosphoglycerate kinase (PGK, spot 63), fructose-bisphosphate aldolase (spot 38), and transketolase (TKT, spot 78). Carbonic anhydrase provides inorganic carbon to improve photosynthetic efficiency and cell resistance to cytotoxic concentrations of H_2_O_2_ under salinity [33], which increased in T1 but decreased in S2 at the late salinity stages (Table 3 and Fig. 2c). Phosphoribulokinase (Fig. 2b) provides an immediate CO_2_ acceptor and is considered the regulatory enzyme of the Calvin cycle [34]. The dissolved CO_2_ is fixed by RuBisCO to produce 3-phosphoglycerates [27]. RuBisCO subunits increased in T1, but the large subunits decreased in S2 (Table 3 and Fig. 2a), which implies higher sensitivity of this subunit to salinity. Transketolase is involved in the regeneration phase of the Calvin cycle and the pentose phosphate pathway, thus influencing plant productivity [35]. This enzyme was upregulated in T1 during the early salt-stress stage, which decreased in the later stages in both genotypes. This suggests that the Calvin cycle in chickpea can be slowed by salinity, and photosynthetic enzymes in S2 are more severely and rapidly impaired than in T1. Similar impairments in carbon assimilation have been reported in proteomic studies in salt-stressed soybean genotypes [23] and H_2_O_2_-stressed rice seedlings [36].

In addition, spot 27 was identified as magnesium chelatase, the first committed enzyme in the chlorophyll biosynthesis pathway [37]. This finding is consistent with our previous studies where salt-stressed T1 recorded higher photosynthesis [38] and biomass [39] than salt-stressed S2. These results suggest that Mg-chelatase is a salt-tolerance factor in T1 by affecting photosynthesis and biomass. The up- and down-regulation of Mg-chelatase after salinity stress has also been reported in barley [40].

In general, the above results suggest that the salinity-tolerant chickpea genotype (T1) withstands salinity through the upregulation of proteins related to light-dependent reactions and the electron transfer pathway (particularly PPD1, CAB, OEE, and ATP synthase). These processes, in turn, provide sufficient capacity and energy equivalents required for the Calvin cycle [22, 23].

### Stress protection by photorespiration

Photorespiration (C_2_ cycle) results from the oxygenase reaction catalyzed by RuBisCO to produce CO_2_ and NH_3_, which wastes ATP and reduction equivalents. Due to this inadequacy, photorespiration may act as an energy sink to inhibit further reductions in the photosynthetic electron transport chain and photoinhibition, which are crucial for plants under stress conditions [41]. Besides, photorespiration generates other metabolites such as serine and glycine for the synthesis of glutathione [42]. Glutathione is an antioxidative factor in plants [42]; accordingly, photorespiration can be involved in stress protection (Fig. 5).

Three DEPs were involved in photorespiration: glycine dehydrogenase (GDH, spot 7), glutamate glyoxylate aminotransferase (GGAT, spot 21), and D-glycerate 3-kinase (GLYK, spot 28). A differential expression pattern was recorded for these DEPs in the two genotypes, which were upregulated at 3 DAT (GDH and GGAT) and 10 DAT (GLYK) in T1 and downregulated at a later stage of stress in S2. Similarly, GDH declined in a drought-sensitive cultivar of *C. dactylon* but increased or remained stable in a tolerant cultivar [43].

Glutamate glyoxylate aminotransferase plays a central role in the biosynthesis and metabolism of nitrogen and certain amino acids, including Gln, Glu, Ser, and Gly. This enzyme catalyzes the phosphorylation of glycerate as the concluding reaction of the C_2_ cycle in chloroplasts [41, 44]. Despite the critical roles of GGAT and GLYK, studies investigating stress responses rarely focus on them. Recently, GGAT was upregulated in faba bean under drought stress and recognized as an important factor in drought tolerance [45]. The elevated abundance of C_2_-cycle-related proteins in T1 under salinity appears to be an essential process in the control of amino acid and glutathione biosynthesis and other metabolisms.

### Defense and detoxification under salinity

Fourteen DEPs were recognized as stress-responsive and implicated in detoxification and defense mechanisms such as ROS production, control of oxidative damage, protein stabilization, and hyperosmotic stress.

#### Heat shock proteins (HSPs) and late embryogenesis abundant (LEA) proteins

The HSP family are high-temperature-inducible chaperones that regulate normal plant growth processes by helping with protein folding and preventing protein aggregation. Three HSP 70 molecular chaperones (spots 1, 11 and 4) and one 20 kDa chaperonin (spot 48) were significantly upregulated in T1, especially at 3 and 10 DAT, respectively (Table 3). However, mitochondrial isoforms (HSP 70, spot 4 and HSP 22, spot 58) progressively increased (spot 4) or decreased (spot 58) during the stress. The higher abundance of chaperones and chaperonin in T1 implies that protein refolding and stabilization might be enhanced to cope with salinity.

In addition, the LEA (spot 30) proteins usually involved in salt tolerance, known as high-molecular osmolytes, function to protect the steady structure of proteins, chlorophyll, membranes, and cells [46]. Upregulation of LEA proteins in T1 could be involved in the process of adaptation to saline conditions (Fig. 5).

#### Antioxidant and detoxifying enzymes

As illustrated in Fig. 5, salinity perturbs water uptake leading to stomatal closure in response to ABA and Ca^2+^, which reduces the entrance of CO_2_, resulting in a net reduction of photosynthesis and subsequent accumulation of ROS with oxidative stress [47, 48]. Beside chloroplasts, ROS are produced in mitochondria, peroxisomes, apoplasts, and their membranes, and involved in aerobic metabolism and active electron transport [35]. In this study, three defense-related enzymes were significantly regulated by salinity. The Cu/Zn superoxide dismutase (spot 77, SOD) and ascorbate peroxidase (spots 41 and 55, APX) were classified in Cluster 5 (Fig. 3c and Additional file 3: Table S1), and upregulated in T1 at 3 DAT and significantly (*p* ≤ 0.05) downregulated at later stages of stress. Glutathione S-transferase (spot 42, GST) had the reverse pattern under salinity and was grouped in Cluster 2. Superoxide dismutase acts as the first line of defense by transforming superoxide into H_2_O_2_, and any excess is removed by the activities of APX [49]. The GST represent a major group of detoxification enzymes in the GPX/GST pathway, which removes H_2_O_2_ [6].

#### Other defensive proteins

Spots 31 and 40 were identified as thiamine thiazole synthase and xanthoxin dehydrogenase, which are involved in the biosynthesis of thiamine [50] and ABA [51], respectively. Thiamine (B1 vitamin) may be involved in DNA damage tolerance in plant cells under oxidative stress [50]. In addition to ABA biosynthesis and regulation, xanthoxin dehydrogenase participates in proline biosynthesis and the sugar-mediated signaling pathway [52]. The elevated levels of these enzymes in T1 at 3 and 6 DAT can protect seedlings against the deleterious effects of ROS.

The cold-shock domain protein (spot 81) may act as an RNA-chaperone in the regulation of translation [53]. It has been induced in the leaves of rice [54] and roots of wheat [55] under salt stress. It is possible that the higher expression of the cold-shock protein in T1 than S2 in the early stages of salinity helps in the translation process by removing secondary structures of mRNA and regulating gene expression by dsDNA interaction.

Apolipoprotein D (ApoD, spot 83), expressed in T1 more than S2 (Table 3), is a small plasma membrane-associated protein known as lipocalin. In Arabidopsis, ApoD has protective functions against oxidative stresses induced by freezing, light, and paraquat [56]. Apolipoprotein D binds and scavenges peroxidated lipids, which helps to maintain membrane integrity. Taken together, the elevated levels of these scavengers, especially in T1 under salinity, are likely strategies for chickpea to cope with the deleterious effects of ROS.

### Protein metabolism under salinity

Proteins associated with metabolism accounted for nearly 17% of DEPs in stressed seedlings (Fig. 3a), and were divided into two functional groups: protein biogenesis and protein degradation. The first group consisted of seven identities including three elongation factor Tu (EF, mitochondrial: spot 13 and chloroplastic: spots 20 and 23), three ribosomal proteins (RP, 30S: spot 24; 50S: spot 85 and 60S: spot 72), and one peptidyl-prolyl cis-trans isomerase (PPIase, spot 16). In response to salt stress, the EFs were significantly upregulated at 1 and 3 DAT, especially in T1, and then sharply downregulated at 10 DAT in both genotypes, particularly S2 (Table 3). Elongation factors contribute to the initiation and elongation of newly growing peptide chains [36], which may explain the enhanced biosynthesis or repair of salt-stressed proteins. In confirming this, 30S RP and PPIase were upregulated at 3 DAT, especially in T1, which indicates T1’s greater capacity for protein biogenesis, folding and stability. A decreased relative abundance of synthetic proteins at later stages of salinity, especially in S2, indicate further suppression of protein biosynthesis in salt-stressed S2. Similar data have been reported in salt-susceptible genotypes of common bean [31] and *Brassica napus* [57] seedlings.

Salinity usually causes protein damage or misfolding, mainly due to ROS accumulation (Fig. 5). The replacement of dysfunctional proteins with newly activated ones is vital during the stress [58]. In this investigation, the initial upregulation of ATP-dependent zinc metalloprotease FTSH 2 (spot 2) and 26S protease regulatory (spot 25) and later increases in metacaspase (spot 60) and proteasome (spot 43), especially in T1 (Table 3), may support this mechanism. The continuous upregulation of FTSH (spot 2; Fig. 2a) in T1, an ATP-dependent metalloprotease, reflects the enhanced replacement of the D1 core component of PSII during salinity [59]. In addition to protease activity, FTSH is a molecular chaperone [60]. Therefore, under salinity FTSH could help to maintain quality control of certain membrane and cytoplasmic proteins, which in turn, could be a salt-tolerance factor in chickpea. However, the observed differential regulation of distinct components in the protein biogenesis and degradation machinery suggests that a complicated mechanism is involved in controlling protein metabolism under salinity, which depends on time and genotype.

### Amino acid and nitrogen metabolism

Our analysis revealed changes in four identities related to the accumulation of organic solutes and nitrogen compounds under salinity (Table 2). The S-adenosylmethionine synthase (SAM-S, spot 19) is a donor of the methyl group in the methylation reactions of proteins, nucleic acids, polysaccharides, and fatty acids, and a precursor for ethylene and polyamine biosynthesis. This enzyme was upregulated in T1 up to 6 DAT but then significantly declined, especially in S2. Higher expression of the SAM-S encoding gene in *Suaeda salsa* [61] and its protein in soybean leaves [23] increased salinity tolerance.

The higher abundance of glutamine synthetase (GS, spot 35) in T1 than S2 produced more glutamate, causing re-assimilation of the extra ammonia released during salinity, and ensuring the production of nitrogenous compounds related to the stress, which can enhance photorespiration to improve stress resistance [45]. Glycine dehydrogenase (GLDC; spot 39) is crucial for the biosynthesis of glycine betaine and facilitates osmotic adjustment [62]. The observed upregulation of GS2 and GLDC in T1 resulted in the accumulation of compatible organic solutes in leaves, which could be linked to salt tolerance. Several studies have also shown that salt stress upregulates GS and GLDC [27].

### Modifications in stress signaling and gene regulation

The signal transduction pathways can regulate gene expression, which leads to the expression of responsive proteins, specifically in the case of abiotic stresses. Spot 47 was characterized as an auxin-binding protein (ABP19a), which notably participates in signal transduction in the presence of abiotic stresses. It functions as an auxin receptor and has an essential role in many developmental processes [63]. The progressively increasing levels of ABP19 over time in T1, relative to S2, suggest an important role of auxin in salinity stress responses. Upregulation of some members of the ABP family has occurred under abiotic stress in maize [23]. To our knowledge, little information is available on ABP members and their roles in response to salinity.

An initial upregulation followed by later downregulation in the expression of the nascent polypeptide-associated complex subunit beta (NAC, spot 82) was observed in both genotypes. This transcriptional reprogramming factor associated with plant stress responses can bind to ribosome-associated nascent polypeptide chains to regulate its sorting and translocation [64]. In response to salt stress, there are reports of NAC upregulated in tomato [65] and downregulated in rice roots [66].

Two ribonucleoproteins (RNP, 33 kDa: spot 37 and 29 kDa: spot 54) that appeared in Cluster 4 (Fig. 3c and Additional file 3: Table S1) are involved in the 3-end processing of chloroplast mRNAs, and their upregulation in T1 may be related to the translation of defense-related genes in the chloroplast [67]. Another RNA processing DEP was a glycine-rich RNA-binding protein (spot 75), which decreased in both genotypes at 10 DAT.

### Other metabolisms

Other proteins associated with the TCA cycle (isocitrate dehydrogenase, IDH, spot 12), oxidative pentose phosphate pathway (6-phosphogluconate dehydrogenase, 6PGDH, spot 15), and purine metabolism (phosphoribosylformylglycinamidine cyclo-ligase, spot 36) were initially upregulated, especially in T1, and then downregulated at 6 and 10 DAT, more so in S2.

Isocitrate dehydrogenase provides NADPH for plants to cope with oxidative stress, and supplies 2-oxoglutarate, which is involved in the glutamine–glutamate synthase cycle. It is crucial, especially in carbon-limiting conditions that experience salinity stress [68]. Similarly, an increase in IDH abundance and the citrate cycle to generate more energy to combat salinity has been reported in tolerant genotypes of rice [69] and wheat [55]. Taken together, an increase in both IDH and 6PGDH abundance in T1 may have accumulated NADPH to supply the necessary energy for salt tolerance.

### Transcriptional investigation

To correlate the levels of identified DEPs with their gene expression patterns, an RT-PCR assay was used to analyze mRNA levels in six corresponding genes (Table 2; Fig. 4; Additional file 5: Figure S4). Five transcripts in T1 (carbonic anhydrase, glycerate dehydrogenase, HSP 70 kDa, ascorbate peroxidase, and phosphogluconate dehydrogenase) and two transcripts in S2 (carbonic anhydrase and HSP 70 kDa) recorded similar trends in their protein profiles. This consistency suggests that these proteins are initially regulated at the transcriptional level, do not misfold or dysfunction after salt treatment, and induce related signal transduction pathways to resist salinity stress [70].

One gene in T1 (metalloprotease) and four in S2 (glycerate dehydrogenase, ascorbate peroxidase, metalloprotease, and phosphogluconate dehydrogenase) displayed different or reversed trends between mRNA and protein levels (Fig. 4; Additional file 5: Figure S4). This inconsistency might be due to the complex mechanisms of protein expression regulations [71] and the presence of multigene families [72]. On the other hand, the parallel and independent changes between protein and mRNA abundance for these genotypes might reflect the complex, mediated regulatory mechanisms of plants in response to salinity [20, 22, 70]. Moreover, correlations between the transcriptome and proteome are not always straightforward and vary due to species, growth stage, and environmental conditions.

## Conclusions

Comparative proteomic analyses using two contrasting chickpea genotypes under control and salt-stressed conditions provided the basis for revealing salt-tolerance mechanisms. Based on our current and previous observations, as well as other reports, a model elucidating the role of proteins and the mechanisms underlying salinity tolerance is depicted in Fig. 5. Salinity stress can be characterized by enhanced ion toxicity and decreased osmotic potential in plants. In response, plants induce signaling events in the plasma membrane that change gene expression and reduce water release due to stomatal closure. The differential salinity response in T1 and S2 could be related to the reprogramming of numerous DEP expression patterns that induce changes in energy metabolism, including photosynthesis, stress-responsive proteins, protein processes, signaling, and energy metabolism. These salinity-tolerance-associated proteins could be key factors that regulate these pathways, including chlorophyll a-b binding protein, oxygen-evolving enhancer protein, ATP synthase, carbonic anhydrase, RuBisCO, HSP and LEA families, ascorbate peroxidase, elongation factor Tu, auxin-binding protein, and ribonucleoproteins. These findings highlight the significance of photosynthesis-related and stress-responsive proteins in the adaptation of chickpea to salinity stress.

Furthermore, hierarchical clustering data revealed key proteins, especially in Clusters 3 and 4, involved in a dynamic network for salt tolerance in chickpea. The higher correlation between some transcripts and their functional proteins, observed in T1, may be important in salt tolerance, and suggests that these proteins are regulated at the transcriptional level in the tolerant genotype and/or less degraded under saline conditions, relative to the sensitive genotype. The proteomic analyses revealed some novel and unexpected proteins not yet reported in chickpea or in relation to salinity.

## Methods

### Plant materials and salinity treatments

In our previous studies, screening of chickpea genotypes for salinity tolerance revealed Flip 97-43c (T1) and Flip 97-196c (S2) as relatively tolerant and susceptible genotypes, respectively [39, 73]. Seeds of these two genotypes were provided by the Research Center for Plant Sciences, Ferdowsi University of Mashhad, Iran. In the current study, the seeds were sterilized with 3% (w/v) sodium hypochlorite and 70% ethanol, rinsed five times with sterilized distilled water and germinated on wet, sterile filter papers in the dark at 25 ± 2 °C for 48 h. Three uniformly germinated seeds were transferred to 3 l pots containing a mixture of field soil and sand (2:1, w/w), and later thinned to two uniform seedlings per pot. The experiment was conducted in a greenhouse (16/8 h, light/dark cycle; 28 °C/18 °C, day/night). Every 3 days at 16:00, each pot was irrigated to maintain the soil moisture content at ~ 70%. Twenty-one-day-old seedlings of each genotype were subjected to 0 and 100 mM NaCl (Sigma, USA) that had been dissolved in distilled water. The pots were arranged in a completely randomized design with three replicates. Fresh leaves were collected after 1, 3, 6, and 10 d for proteomics and 1, 3 and 5 d for gene expression experiments to extract protein and mRNA, respectively, then frozen in liquid nitrogen and stored at − 80 °C. The choice of NaCl concentration and stress time-points was based on our previous studies, and other proteomics and gene expression analysis reports [6, 73].

### Protein extraction and concentration assay

Total protein extraction was performed using the method of Goggin [74]. Briefly, 1 g of leaves was ground to a fine powder in liquid nitrogen, and placed in two 2 ml tubes with two volumes of extraction buffer containing 7 M urea, 2 M thiourea, 2% (v/v) Triton X-100, 20 mM DTT, and 4% (w/v) CHAPS. After 20 min incubation on ice with gentle rocking, the tubes were centrifuged at 12,000 *g* for 10 min. For purification, the supernatant was precipitated in 9 ml chilled acetone (− 80 °C), incubated for 1 h at − 80 °C, then centrifuged at 14,000 *g* for 30 min. The green supernatant was removed. The pellet was washed with 5 ml chilled acetone, dried at room temperature, and resuspended in minimal IEF buffer containing 7 M urea, 2 M thiourea, 4% (w/v) CHAPS, 60 mM DTT, 2% (v/v) immobilized pH gradient (IPG) buffer (pH 4–7) for 10 min with gentle rocking. All centrifugations were carried out at 2 °C. The protein concentration in the supernatant was determined by Bradford assay using crystalline BSA as standard [75].

### Two-dimensional polyacrylamide gel electrophoresis (2D-PAGE)

2D-PAGE of protein samples was carried out according to the method of O’Farrell [76]. In brief, IPG strips (pH 4–7 linear, 17 cm, BioRad) were loaded with 350 μl 2-DE rehydration buffer containing 500 μg protein and, after 1 h, covered with 2 ml mineral oil. The passive rehydration step was performed at room temperature for 16 h, then IEF was carried out with a PROTEAN IEF Cell system (BioRad, USA) under the following conditions: 1 h at 300 V, gradient from 300 V to 6000 V over 2 h, and hold at 6000 V for 6 h (total 40,000 kVh). The current was 50 μA per strip, and the temperature was maintained at 20 °C. The focused IPG strips were reduced in 10 ml equilibrium buffer (6 M urea, 50 mM Tris pH 8.8, 30% [v/v] glycerol, 2% [w/v] SDS and 0.002% [w/v] bromophenol blue) with gentle shaking containing 65 mM DTT (Sigma, USA) for 15 min. Then, the strips were placed in the same buffer for alkylation containing 135 mM iodoacetamide (Sigma, USA) for 15 min, followed by brief washing in 1× SDS–Tris–glycine running buffer, as described by Chivasa et al. [77].

For second dimension separation of proteins, the developed IPG strips were applied to SDS-PAGE gels containing 12.5% (w/v) polyacrylamide (Sigma, USA) using a PROTEAN II Multi Cell (BioRad, USA), with protein markers (Fermentas, Waltham, MA, USA) loaded on the left side and overlaid with agarose solution. The electrophoresis was run at 15 mA per gel for 30 min, subsequently increased to 30 mA/gel for 8 h. Following SDS-PAGE, gels were washed in ddH_2_O and stained with 0.12% (w/v) Coomassie Brilliant Blue G-250 solution followed by destaining in 1% (v/v) acetic acid as described by Candiano et al. [78].

### Image acquisition and statistical analysis

The gels were scanned using a GS-800 calibrated densitometer (BioRad) at 600 dpi resolution. Spot detection, spot measurement, background subtraction, and spot matching were conducted using Image Master 2D Platinum (ver. 6.01). Following automatic spot detection and matching, all gel images were double-checked manually. To normalize spot volumes, the quantitative amount of protein spots was expressed as percent volumes (percentage of total volume in all spots in the gel) to overcome experimental errors introduced due to differential staining. The molecular mass (*M*_r_) and isoelectric pH (pI) of proteins were calculated on digitized gels using standard protein markers (Sigma, USA) and interpolation of values on the IPGs, respectively.

Significant differences between means were calculated by SPSS software (ver. 24) using one-way analysis of variance and Duncan’s multiple range test. Spots that were consistently present in all three replicates gels and recorded statistically significant (*p* ≤ 0.05) changes between time-points or relative to their respective control were accepted for further analysis. These spots were then filtered, according to at least two fold-changes in the expression. The data were expressed as fold-changes that were calculated by an increase (+) or decrease (−) of percent volume in each protein spot at each time-point relative to the same protein spot in the control.

### Protein identification and database searching

Selected protein spots were excised from 2-DE gels and digested with trypsin, and the peptides were extracted based on standard techniques [79] at Proteomics International, Nedlands, Western Australia. Protein spots were identified using liquid chromatography coupled with tandem mass spectrometry (LC-MS/MS) with an Agilent 1260 Infinity HPLC system (Agilent) coupled to an Agilent 1260 Chipcube Nanospray interface (Agilent) on an Agilent 6540 mass spectrometer (Agilent). Peptides were loaded onto a ProtID-Chip-150 C18 column (Agilent) and separated with a linear gradient of water/acetonitrile/0.1% formic acid (v/v). To identify proteins of interest, the raw spectra were submitted to the MSPnr100 database search using MASCOT sequence matching software (Matrix Science, London, UK; http://www.matrixscience.com). Database interrogation parameters for LC-MS/MS analysis on the Agilent 6540 mass spectrometer were as follows: taxonomy (Viridiplantae: green plants), variable modifications (carbamidomethyl, oxidation of methionine residues), mass values (monoisotopic), protein mass (unrestricted), peptide mass tolerance (±0.2 Da), MS/MS tolerance (± 0.2 Da), peptide charge state (2+, 3+ and 4+), enzyme (trypsin), and maximum missed cleavages (1). Only significant hits, as defined by the MASCOT probability analysis (*p* ≤ 0.05) were accepted.

### Functional classification and clustering analysis

The identified proteins were grouped into gene ontology categories and mapped based on their putative functional and likely metabolic roles by investigations in Blast2GO software and databases as Kyoto Encyclopedia of Genes and Genomes (https://www.genome.jp), NCBI and UniProt [80]. Furthermore, literature reviews were used, if available, to confirm biological processes.

The co-expression pattern of the identified proteins was specified using a self-organizing tree algorithm (SOTA) hierarchical clustering. The changes in fold expression values across four time-points (t1–t4) were log transformed, and clustering was undertaken with Pearson correlation as distance with 10 cycles and a maximum cell diversity of 0.8, using Multi-Experimental Viewer (The Institute for Genomic Research) version 4.5.1 [81].

### RNA extraction, cDNA synthesis, and semi-quantitative RT-PCR

Total RNA was extracted from the leaves in three biological replicates at three time-points (1, 3 and 5 days after salt treatment [DAT]) using RNeasy Plant Mini Kit (Qiagen, Germany), and treated with RNase-Free DNase Set (Qiagen, Germany) for further purification, in accordance with the manufacturer’s instructions. The extracted RNA integrity and concentrations were assessed by gel electrophoresis (1.5% (w/v) agarose) and a NanoDrop spectrophotometer (Thermo NanoDrop 2000 UV-vis). The cDNAs were generated from each sample using gene-specific primers (Table 2) by Maxima First Strand cDNA Synthesis kit (Fermentas, Waltham, MA, USA) as follows: 6 μl of DEPC water, 1 μl of 100 ng/μl RNA, 2 μl of 5 × Reaction Mix, and 1 μl of Max Enzyme. The mixture was incubated for 10 min at 25 °C, 15 min at 50 °C, and 5 min at 85 °C.

The PCR reactions were performed in a 25 μl reaction volume containing 1–2 μl of the cDNA as template, 1 μM of each primer, 200 μM of dNTP, 1 unit of Taq polymerase enzyme, and 1 × PCR buffer with 1.5 mM MgCl_2_. A pre-test was conducted with 25, 28, 31, and 34 PCR cycles to determine the optimal number of cycles for amplification of selected genes. The amplification at cycle 28 was in the exponential range and had not reached a plateau yet (Additional file 4: Figure S3). The RT-PCR conditions consisted of an initial incubation of 95 °C for 7 min, followed by 28 cycles of 95 °C for 20 s, annealing at 50–60 °C for 25 s, extension at 72 °C for 30 s, ending with a 7-min incubation at 72 °C.

To design RT-PCR primers, the sequence of each differentially expressed protein (DEP) was first used as a tBLASTn search term against chickpea expressed sequence tags (ESTs). The best aligned EST, along with its corresponding UniGene, were selected; all ESTs clustering with this UniGene were electronically assembled, and the assembled sequence with emphasis on the part of the sequence which contained the identified peptides, was used for primer design using Primer Premier 5. The primer sequences and other features for the RT-PCR assays are in Table 2.

## Additional files

Additional file 1: **Figure S1.** Leaf proteome of T1, a salt-tolerant chickpea genotype after (A) 1, (B) 3, (C) 6, and (A) 10 days of 100 mM NaCl stress. An equal amount (500 μg) of protein from all samples was resolved by 2-DE. The experiment was performed in three replicates and the gels of unstressed seedlings (control) are not shown. (DOCX 387 kb)

Additional file 2: **Figure S2.** Leaf proteome of S2, a salt-susceptible chickpea genotype after (A) 1, (B) 3, (C) 6, and (A) 10 days of 100 mM NaCl stress. An equal amount (500 μg) of protein from all samples was resolved by 2-DE. The experiment was performed in three replicates and the gels of unstressed seedlings (control) are not shown. (DOCX 5332 kb)

Additional file 3: **Table S1.** Classification of different proteins represented in salinity-stressed chickpea seedlings compared to their respective controls based on their expression patterns. (DOCX 20 kb)

Additional file4: **Figure S3.** Determination of the optimal number of PCR cycles for selected gene amplification. (DOCX 105 kb)

Additional file 5: **Figure S4.** The effects of 100 mM salt stress for 1, 3, and 5 days on mRNA expressions, and 1, 3, 6, and 10 days of protein changes in abundance of (A) carbonic anhydrase, (B) glycerate dehydrogenase, (C) heat shock 70 kDa protein, (D) L-ascorbate peroxidase, (E) zinc metalloprotease FTSH2, and (F) phosphogluconate dehydrogenase in the seedling leaves of chickpea genotypes T1 and S2. Transcript levels were determined by RT-PCR, using the chickpea actin gene as a control for normalization, and expressed as fold changes (increase or decrease) relative to the respective control. Data represents the mean of three biological replicates and the vertical bars indicate ±SE. (DOCX 287 kb)

## Abbreviations

2-DE: Two-dimensional electrophoreses
6PGDH: 6-phosphogluconate dehydrogenase
ABP: Auxin-binding protein
ApoD: Apolipoprotein D
APX: Ascorbate peroxidase
CA: Carbonic anhydrase
CAB: Chlorophyll a-b binding protein 3
DAT: Days after salt treatment
DEPs: Differentially expressed proteins
ECM: Extracellular matrix
EF: Elongation factor Tu
FBPA: Fructose-bisphosphate aldolase
GDH: Glycine dehydrogenase
GGAT: Glutamate glyoxylate aminotransferase
GLDC: Glycine dehydrogenase
GLYKD: Glycerate 3-kinase
GS: Glutamine synthetase
GST: S-transferase
HSPs: Heat shock proteins
IDH: Isocitrate dehydrogenase
LC-MS/MS: Liquid chromatography–mass spectrometry
LEA: Late embryogenesis abundant
MAPK: Mitogen-activated protein kinase
*M*_r_: Molecular mass
NAC: Nascent polypeptide-associated complex subunit beta
OEE: Oxygen-evolving enhancer protein
PGK: Phosphoglycerate kinase
p*I*: Isoelectric pH
PPD1: PsbP domain-containing protein 1
PPIase: Peptidyl-prolyl cis-trans isomerase
PRK: Phosphoribulokinase
PS: Photosystem
PTM: Post-translational modification
RNP: Ribonucleoproteins
ROS: Reactive oxygen species
RP: Ribosomal protein
RuBisCO: Ribulose 1,5-bisphosphate carboxylase/oxygenase
SAM-S: S-adenosylmethionine synthase
SOD: Cu/Zn superoxide dismutase
SOTA: Self-organizing tree algorithm
TKT: Transketolase
