# Supplementary material for: Comparative proteomics and gene expression analyses revealed responsive proteins and mechanisms for salt tolerance in chickpea genotypes
Source: BMC Plant Biol. 2019 Jul 9;19:300. doi: 10.1186/s12870-019-1793-z (PMC6617847; doi:10.1186/s12870-019-1793-z)
Supplement: Supplementary file 1 — Figure S1. Leaf proteome of T1, a salt-tolerant chickpea genotype after (A) 1, (B) 3, (C) 6, and (A) 10 days of 100 mM NaCl stress. An equal amount (500 μg) of protein from all samples was resolved by 2-DE. The experiment was performed in three replicates and the gels of unstressed seedlings (control) are not shown. (DOCX 387 kb) [file 12870_2019_1793_MOESM1_ESM.docx]

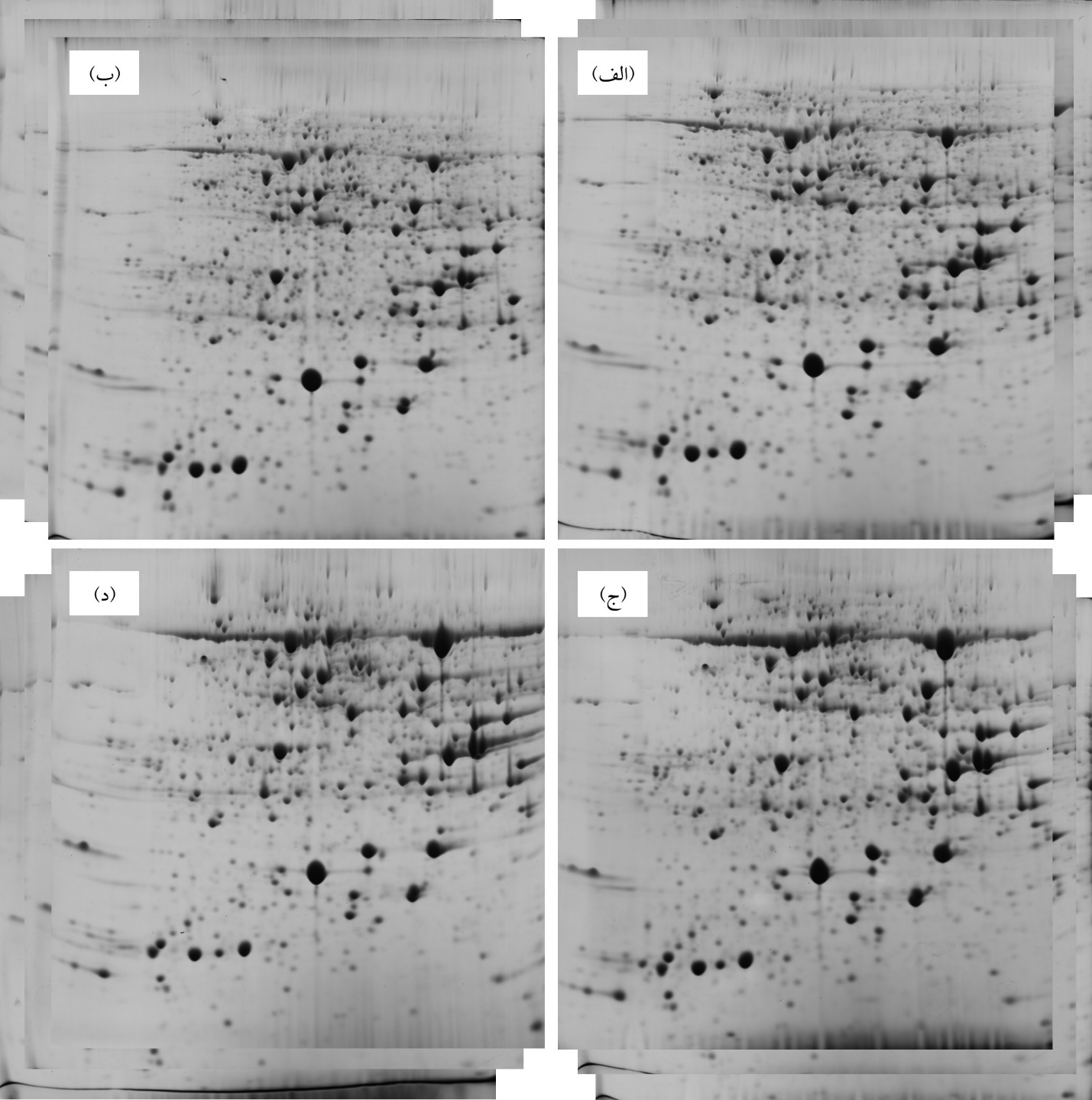


(A)

(D)

(C)

(B)

**Additional file 1: Figure S1.** Leaf proteome of T1, a salt-tolerant chickpea genotype after (A) 1, (B) 3, (C) 6, and (A) 10 days of 100 mM NaCl stress. An equal amount (500 µg) of protein from all samples was resolved by 2-DE. The experiment was performed in three replicates and the gels of unstressed seedlings (control) are not shown.
