# Supplementary material for: Comparative proteomics and gene expression analyses revealed responsive proteins and mechanisms for salt tolerance in chickpea genotypes
Source: BMC Plant Biol. 2019 Jul 9;19:300. doi: 10.1186/s12870-019-1793-z (PMC6617847; doi:10.1186/s12870-019-1793-z)
Supplement: Supplementary file 3 — Table S1. Classification of different proteins represented in salinity-stressed chickpea seedlings compared to their respective controls based on their expression patterns. (DOCX 20 kb) [file 12870_2019_1793_MOESM3_ESM.docx]

**Additional file 3: Table S1.** Classification of different proteins represented in salinity-stressed chickpea seedlings compared to their respective controls based on their expression patterns.

| **Spot no.** | **Identified protein** | **Cluster** | **Function** |
| --- | --- | --- | --- |
|  | **Pattern 1 (n=4)** |  |  |
| 43 | Proteasome subunit beta type | 1 | Protein synthesis and degradation |
| 60 | Metacaspase-4-like | 1 | Protein synthesis and degradation |
| 42 | Glutathione s-transferase | 2 | Stress responsive |
| 84 | Ribulose bisphosphate carboxylase small chain | 2 | Photosynthesis and bioenergy |
|  | **Pattern 2 (n=28)** |  |  |
| 04 | Heat shock 70 kDa protein, mitochondrial isoform | 3 | Stress responsive |
| 28 | D-glycerate 3-kinase, chloroplastic | 3 | Photorespiration |
| 30 | uncharacterized protein, Homologues with LEA-2 | 3 | Stress responsive |
| 39 | Glycerate dehydrogenase | 3 | Amino acid and nitrogen metabolism |
| 47 | Auxin-binding protein ABP19a-like | 3 | Signaling |
| 01 | 70 kDa heat shock protein | 4 | Stress responsive |
| 02 | ATP-dependent zinc metalloprotease FTSH 2 | 4 | Protein synthesis and degradation |
| 03 | RuBisCO large subunit-binding protein subunit beta | 4 | Photosynthesis and bioenergy |
| 15 | 6-phosphogluconate dehydrogenase, decarboxylating 3 | 4 | Other metabolisms (PP) |
| 16 | peptidyl-prolyl cis-trans isomerase CYP38, chloroplastic | 4 | Protein synthesis and degradation |
| 18 | ATP synthase beta subunit, partial (chloroplast) | 4 | Photosynthesis and bioenergy |
| 21 | Glutamate--glyoxylate aminotransferase | 4 | Photorespiration |
| 24 | 30S ribosomal protein S1, chloroplastic | 4 | Protein synthesis and degradation |
| 25 | 26S protease regulatory subunit 6A homolog | 4 | Protein synthesis and degradation |
| 26 | Phosphoribulokinase, chloroplastic | 4 | Photosynthesis and bioenergy |
| 27 | Magnesium-chelatase subunit ChlI, chloroplastic-like | 4 | Photosynthesis and bioenergy |
| 33 | ATP synthase subunit beta, chloroplastic | 4 | Photosynthesis and bioenergy |
| 35 | Glutamine synthetase leaf isozyme, chloroplastic | 4 | Amino acid and nitrogen metabolism |
| 37 | 33 kDa ribonucleoprotein, chloroplastic | 4 | Gene transcription and replication |
| 38 | Fructose-bisphosphate aldolase 1, chloroplastic | 4 | Photosynthesis and bioenergy |
| 40 | Xanthoxin dehydrogenase | 4 | Stress responsive |
| 45 | Carbonic anhydrase, chloroplastic isoform X2 | 4 | Photosynthesis and bioenergy |
| 48 | 20 kDa chaperonin, chloroplastic-like | 4 | Stress responsive |
| 49 | psbP domain-containing protein 1, chloroplastic | 4 | Photosynthesis and bioenergy |
| 52 | Chlorophyll a-b binding protein 3, chloroplastic | 4 | Photosynthesis and bioenergy |
| 53 | ATP synthase subunit alpha | 4 | Photosynthesis and bioenergy |
| 54 | 29 kDa ribonucleoprotein A, chloroplastic | 4 | Gene transcription and replication |
| 58 | Heat shock 22 kDa protein, mitochondrial isoform X1 | 4 | Stress responsive |
|  | **Pattern 3 (n=32)** |  |  |
| 13 | Elongation factor Tu, mitochondrial | 5 | Protein synthesis and degradation |
| 20 | Elongation factor Tu, chloroplastic | 5 | Protein synthesis and degradation |
| 55 | L-ascorbate peroxidase, cytosolic | 5 | Stress responsive |
| 63 | Phosphoglycerate kinase, cytosolic-like | 5 | Photosynthesis and bioenergy |
| 64 | Oxygen-evolving enhancer protein 1, chloroplastic | 5 | Photosynthesis and bioenergy |
| 65 | smad/FHA domain protein | 5 | Gene transcription and replication |
| 68 | 2Fe-2S iron-sulfur cluster-binding domain protein | 5 | Photosynthesis and bioenergy |
| 69 | Cytochrome b6-f complex iron-sulfur subunit | 5 | Photosynthesis and bioenergy |
| 71 | Histone H2B | 5 | Gene transcription and replication |
| 74 | Photosystem I reaction center subunit II, chloroplastic-like | 5 | Photosynthesis and bioenergy |
| 75 | Glycine-rich RNA-binding protein-like | 5 | Gene transcription and replication |
| 77 | Superoxide dismutase [Cu-Zn] | 5 | Stress responsive |
| 78 | Transketolase, chloroplastic | 5 | Photosynthesis and bioenergy |
| 79 | Oxygen-evolving enhancer protein 2, chloroplastic | 5 | Photosynthesis and bioenergy |
| 81 | Cold shock protein | 5 | Stress responsive |
| 82 | Nascent polypeptide-associated complex subunit beta | 5 | Gene transcription and replication |
| 85 | 50S ribosomal protein L9, chloroplastic | 5 | Protein synthesis and degradation |
| 06 | Probable mitochondrial-processing peptidase subunit beta | 6 | Protein synthesis and degradation |
| 07 | Glycine dehydrogenase (decarboxylating), mitochondrial | 6 | Photorespiration |
| 11 | Heat shock protein 70 | 6 | Stress responsive |
| 12 | Isocitrate dehydrogenase [NADP] | 6 | Other metabolisms (TCA) |
| 14 | Polyadenylate-binding protein RBP45B isoform X2 | 6 | Gene transcription and replication |
| 19 | S-adenosylmethionine synthase | 6 | Amino acid and nitrogen metabolism |
| 23 | Elongation factor Tu, chloroplastic | 6 | Protein synthesis and degradation |
| 31 | Thiamine thiazole synthase, chloroplastic | 6 | Stress responsive |
| 36 | Phosphoribosylformylglycinamidine cyclo-ligase, chloroplastic/mitochondrial-like | 6 | Other metabolisms (purine) |
| 41 | L-ascorbate peroxidase, cytosolic | 6 | Stress responsive |
| 46 | Hydroxyacylglutathione hydrolase 2, mitochondrial-like isoform X | 6 | Amino acid and nitrogen metabolism |
| 70 | Ribulose-1,5-bisphosphate carboxylase/oxygenase large subunit, partial (chloroplast) 2 | 6 | Photosynthesis and bioenergy |
| 72 | 60S acidic ribosomal protein P3-like | 6 | Protein synthesis and degradation |
| 80 | Low molecular weight phosphotyrosine protein phosphatase | 6 | Signaling |
| 83 | Apolipoprotein D-like | 6 | Stress responsive |
