# Supplementary material for: Comparative proteomics and gene expression analyses revealed responsive proteins and mechanisms for salt tolerance in chickpea genotypes
Source: BMC Plant Biol. 2019 Jul 9;19:300. doi: 10.1186/s12870-019-1793-z (PMC6617847; doi:10.1186/s12870-019-1793-z)
Supplement: Supplementary file 4 — Figure S3. Determination of the optimal number of PCR cycles for selected gene amplification. (DOCX 105 kb) [file 12870_2019_1793_MOESM4_ESM.docx]

*Actin*

*Carbonic anhydrase* (45)

*Glycerate dehydrogenase* (39)

*Heat shock 70 kDa protein* (4)

*L-ascorbate peroxidase* (55)

*ATP-dependent zinc metalloprotease FTSH2* (2)

*6-phosphogluconate dehydrogenase* (15)


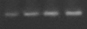

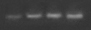

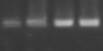

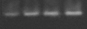

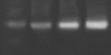


25 28 31 34

PCR cycles

Gene (**Spot no.**)


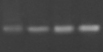

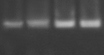

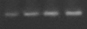

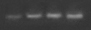

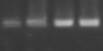

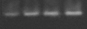

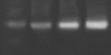

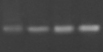

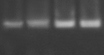


**Additional file 4: Figure S3.** Determination of the optimal number of PCR cycles for selected gene amplification.
