# Supplementary material for: Comparative proteomics and gene expression analyses revealed responsive proteins and mechanisms for salt tolerance in chickpea genotypes
Source: BMC Plant Biol. 2019 Jul 9;19:300. doi: 10.1186/s12870-019-1793-z (PMC6617847; doi:10.1186/s12870-019-1793-z)
Supplement: Supplementary file 5 — Figure S4. The effects of 100 mM salt stress for 1, 3, and 5 days on mRNA expressions, and 1, 3, 6, and 10 days of protein changes in abundance of (A) carbonic anhydrase, (B) glycerate dehydrogenase, (C) heat shock 70 kDa protein, (D) L-ascorbate peroxidase, (E) zinc metalloprotease FTSH2, and (F) phosphogluconate dehydrogenase in the seedling leaves of chickpea genotypes T1 and S2. Transcript levels were determined by RT-PCR, using the chickpea actin gene as a control for normalization, and expressed as fold changes (increase or decrease) relative to the respective control. Data represents the mean of three biological replicates and the vertical bars indicate ±SE. (DOCX 287 kb) [file 12870_2019_1793_MOESM5_ESM.docx]

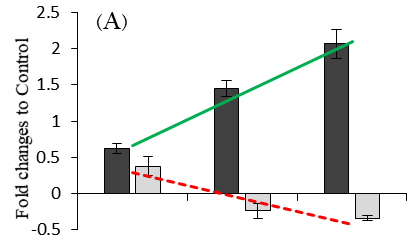

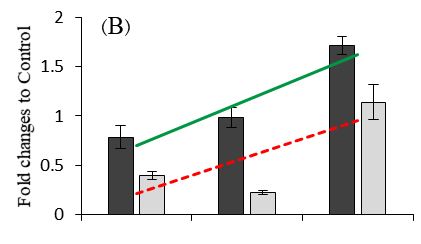

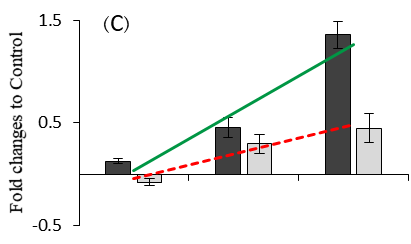

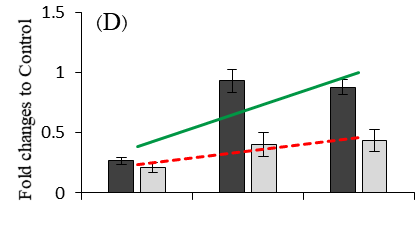

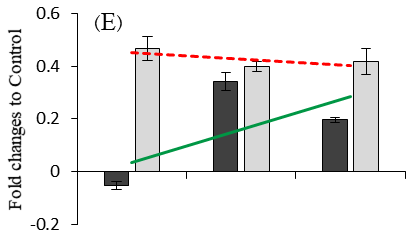

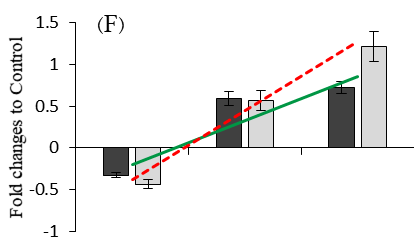


1 3 5

Days after salinity


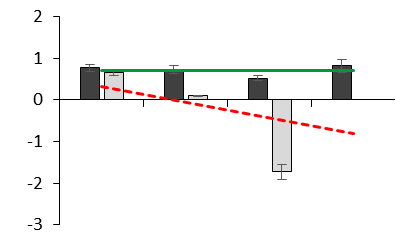

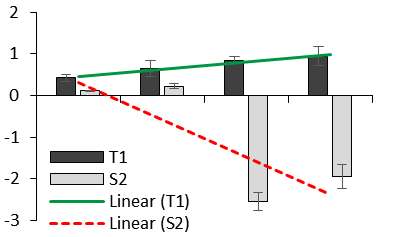

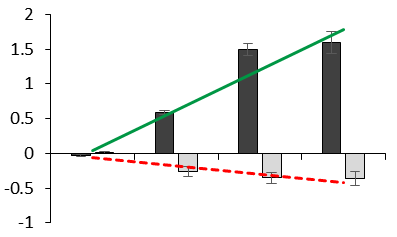

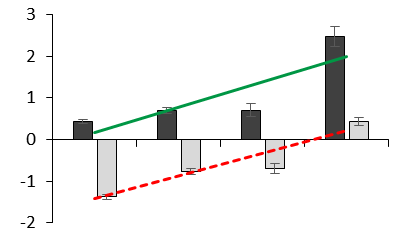

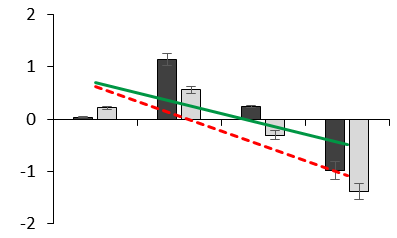

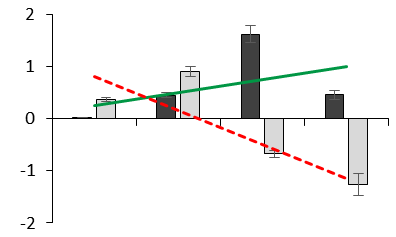


1 3 6 10

Days after salinity

Gene Protein

**Additional file 5: Figure S4.**The effects of 100 mM salt stress for 1, 3, and 5 days on mRNA expressions, and 1, 3, 6, and 10 days of protein changes in abundance of (A) *carbonic anhydrase*, (B) *glycerate dehydrogenase,* (C) *heat shock 70 kDa protein*, (D) *L-ascorbate peroxidase*, (E) *zinc metalloprotease FTSH2*, and (F) *phosphogluconate dehydrogenase* in the seedling leaves of chickpea genotypes T1 and S2. Transcript levels were determined by RT-PCR, using the chickpea actin gene as a control for normalization, and expressed as fold changes (increase or decrease) relative to the respective control. Data represents the mean of three biological replicates and the vertical bars indicate ±SE.
